# Should we evoke fear and responsibility in management of obesity-related risk in the press?

**DOI:** 10.3389/fpsyg.2022.1068464

**Published:** 2023-01-09

**Authors:** Jing Zhao, Xinmin Zheng

**Affiliations:** ^1^School of Foreign Languages, Fuzhou University, Fuzhou, Fujian, China; ^2^Department of Foreign Languages, Zhicheng College, Fuzhou University, Fuzhou, Fujian, China; ^3^School of Education, Shanghai International Studies University, Shanghai, China

**Keywords:** fear, responsibility, obesity, risk, discourse, corpus linguistics, press

## Introduction

Obesity has been considered to be a vital risk to people's health in the modern world, which arouses great anxiety among individuals, medical staff, and governance (Wexler, 2005; Segel, 2011; De Pergola and Silvestris, 2013; Malik et al., 2013; Fruh, 2017). It is obvious that people are influenced consciously or unconsciously by the press when searching for strategies to improve their health. But probably they tend to be filled with fear and guilty rather than confidence and hope as a result. To some extent, it is acknowledged that the obesity risk in media representation has been explored by Lawrence (2004), Kwan and Graves (2013), and Atanasova and Koteyko (2017). Lawrence focuses on the framing contest between personal responsibility and social environment factors. Following Lawrence, Kwan and Graves work on four categories: aesthetic, health, choice and responsibility, and social justice frames and analyze the internal contradictions within a frame and differences and similarities across frames in the production and distribution of social knowledge. Atanasova and Koteyko carry out a comparative study of how British and German newspapers construct obesity as risky. Nonetheless, more studies are needed to dig deep into how the concept of risk is related to obesity in media representation, what differences are existing between different newspapers, and what changes have taken place over time.

With great eagerness, we read the article entitled “Fear and Responsibility: Discourses of Obesity and Risk in the UK Press” by Brookes and Baker (2021a), which examines how the UK print media has represented notions of risk in the coverage of obesity. Specifically, it concentrates upon references to risks related to obesity within the wider context occurring between 2008 and 2017 (inclusive) by using corpus linguistics methods combined with qualitative discourse analysis. As we see it, the article has revealed tricky connotations of fear and responsibility embedded in the newspaper coverage of obesity-related risks. Initially, the corpus-based approach to discourse analysis used as a method in the research is well-presented so that other relevant studies can duplicate the procedures in terms of three techniques, i.e., searching for keywords, doing collocation analysis, and concordance analysis. In addition, a post-structuralist view of discourse based on Foucault's observation of the relation of power and discourse (1969) has been employed, which offers a helpful analytical strategy for qualitative studies to investigate the ideology hidden in discourses. Therefore, we believe the research findings and interpretations are informative and revelatory for practitioners, researchers, and common readers.

Prior to commenting upon the article in detail, it is necessary to straighten up two points mentioned above, namely, the dialectical relationship of media coverage and social life and the method of corpus linguistics employed in obesity risk discourse analysis, so as to help better understand and interpret the article.

The influence of media discourse on society has been extensively studied in critical discourse analysis. Particular attention is paid to the dialectical relationship between discourse constructed in the press and the social practice in everyday life. Fairclough (1995) first applies his critical discourse analysis framework to media discourse to study the relation of power and discourse in the context of social changes, exploring the tensions between public and private, information and entertainment in the press. Since then, researchers (Kress and Van Leeuwen, 2001; Thurlow and Mroczek, 2011) have developed the field of media discourse analysis in a wider sphere, covering different formats, such as multimodal discourse and new media, and different thematic interests are explored, such as commercial, cultural, racial, sexist, and political discourse (Machin and Van Leeuwen, 2007; Talbot, 2007; Triandafyllidou and Wodak, 2009; Johnson and Milani, 2010; Baker et al., 2013; Berger, 2016). However, there are very few studies on the role that the press plays in framing obesity-related risk, which is the very focus of the article.

Another point we would like to look at is the corpus linguistics method used in “obesity and risk” discourse analysis in media. The large collection of British national newspaper articles about obesity published between 2008 and 2017 (inclusive) offers a chance to identify comprehensively and exhaustively discourses through which notions of obesity-related risk and certain “truth” on risk are established and taken as the basis for obesity action (Lupton, 2013). With a discourse-based approach and corpus linguistic methods (Baker, 2006), this article explores how obesity-risk discourses change in two areas: (a) over time and (b) with different types of newspapers. Hence, we think it is necessary to examine how fear and responsibility notions are represented in obesity risk discourses in media coverage based on large data from the national newspapers in the UK over a decade.

## The study

As mentioned in the introduction, the article (Brookes and Baker, 2021a) examines the impact of the UK newspapers on the relationship between obesity and risk based on corpus linguistic analysis. In this section, a summary of this article is made focusing on its analytical framework, method of study, and findings for our further discussion.

The analytical framework of this study is a post-structuralist view of discourse inspired by Foucault (1969) who insists on the power of discourses to construct objects, developed by Lupton (2013) who explains how discourses construct risk as a social phenomenon and disseminate it. Drawing on the social constructionist perspective, this study focuses on a large data study of different national newspapers from 2008 to 2017, attempting to promote the study regarding the obesity risk discourse analysis dimension that has been particularly overlooked and under-theorized (Brookes and Baker, 2021a).

In terms of the research methodology, the study adopts a corpus-based approach to discourse analysis, combining qualitative and quantitative approaches to investigate a decade of newspaper articles on obesity risk. The data are collected from the online news archive *LexisNexis* containing at least one mention of obese or obesity (43,878 articles with 36,053,221 words and 822 words for mean article length in total) and built into a corpus. In addition, four sections of sub-corpora are made according to left-leaning broadsheets, right-leaning broadsheets, left-leaning tabloids, and right-leaning tabloids. An online analysis tool *CQPweb* by Hardie (2012) provides the space to store the corpus and methods to identify the keywords, collocation, and concordance for discourse analysis. Consequently, a qualitative analysis is combined with the words and associations between words that might have been impossible to spot using hand and eye methods. Data analysis is conducted in three stages. First, the data are coded where the risk is described as relevant to obesity; second, the main trends of change over time and in different newspapers have been noted; and third, this analysis takes a holistic consideration of economic, social, historical, and political factors concerning the contexts of relevant texts. In addition, newspaper readerships, the recent popularity of online news sites, and government policies are taken with reference to obesity risk.

The research findings of this article illustrate that there is a prominent difference between the broadsheets and the tabloids based on a large collection of obesity-related risk discourses in British newspaper articles. The former tends to describe the risk factors that may lead to obesity, while the latter stresses the risks of health problems caused by obesity. Another distinction occurs between left-leaning broadsheets that tend to emphasize the social and political determinants of obesity risk and right-leaning broadsheets that call on individuals' responsibility to reduce obesity risk by exercising and adjusting their eating habits. Thus, except for the left-leaning broadsheets, all other newspapers foreground a discourse of individual responsibility for obesity risk.

Therefore, with solid evidence, this study examines profoundly and thoroughly that readers' choice of newspaper determines what messages concerning obesity-related risks and risk management they are exposed to. This study attributes this to “structure violence” (Sakellariou and Rotarou, 2017), which deprives part of the population of the material resources to enact the type of lifestyle changes that newspapers implore them to reduce their risk. Furthermore, what intersects with such neoliberal discourses is the scare tactics which probably make the people who do not possess the social and economic resources feel guilty, angry, and defensive (Hastings et al., 2004), though it is likely to work with individuals who do. As a result, the prominent increase in discursive framing of risk relevant to obesity as dangerous, personalized, and legitimated by findings of scientific research has been witnessed over time. Apparently, even though this study does not dismiss the effectiveness of scare tactics, it holds vital implications for emphasizing reward over risk in motivating readers to manage their eating habits and doing exercises to prevent obesity-related risks.

## Discussion

This article contributes an interesting perspective with convincing empirical evidence for readers to understand how the UK newspapers have manipulated readers' fear and responsibilities in the management of obesity and relevant risks. In this section, we would make comments on the article from the perspective of the selection of data and analytical process for the purpose of promoting future studies. Also, we encourage further studies on other variables concerning media coverage of obesity and risk.

First, in regard to the selection of data resources, the newspapers collected in the case study are all national newspapers, most of which have a wide circulation over the country. According to the article, the authors claim that their research is based on “a corpus consisting of the text from a decade of newspaper articles about obesity from the UK national press” (Brookes and Baker, 2021a: p. 3). It is obvious that national newspapers from 2008 to 2017 are what they are looking at. On the one hand, national newspapers cover a large range of readers from all walks of life and from diverse regions and ethnic groups, the systematic processing of the data and the rigorous analysis are quite convincing. Different categories of newspapers' political tendencies are reflected in their different obesity-risk coverage. On the other hand, as far as we are concerned, we still think it worthwhile to include some local newspapers to make a comparison and contrast to see whether there are certain distinctions between various regions, religious groups, and age groups. As everybody knows, local newspapers are important access for their target readers to exclusive community resources. Therefore, they would cater to their readers' heterogeneous interests and concerns side by side. As Atanasova and Koteyko (2017) point out that a comparative approach in a more in-depth sense will shed more light on different discursive framing strategies and diversified cultures [refer also Huang and Bisiada (2021) for practice in other cultures].

In addition, it is stated that the corpus is divided into four sub-corpora: broadsheet-left, broadsheet-right, tabloid left, and tabloid right. *Guardian* and *Independent* are categorized into broadsheet left; *Telegraph* and *Times* into broadsheet right; *Mirror* tabloid left; and *Express, Mail, Star*, and *Sun* tabloid right. This divide illustrates that the influencing factors concerning obesity-related risks are distinct between broadsheets and tabloids, left and right. We notice, however, this article does not clarify the criteria to categorize the 10 newspapers into four sections. We know that the *Independent* has sometimes claimed itself as center or liberal, and this article finds it less critical of the government (Brookes and Baker, 2021a) compared with the *Guardian*, but they are grouped together into broadsheets left in the article. The presupposition of this grouping analysis implies obviously that the broadsheet differs from the tabloid, left from right in their coverage of obesity-related risks, and the newspapers in the same category seem to share the same political attitudes, which seems a little bit arbitrary. Though the researchers claim that the result echoes their more comprehensive study (Brookes and Baker, 2021b), it may arouse questions about the distinctions in each newspaper's practice. In order to eliminate the blurring images, we suggest each newspaper's data be summarized and demonstrated separately before being categorized into certain political groups.

Second, readers usually get access to several different newspapers for diversified information, such as political affairs, health issues, or risk management, which is particularly convenient for online newspaper websites. Therefore, future studies can conduct a more penetrating investigation by conducting interviews or surveys to explicate the facts from the reception dimension, instead of relying mainly on the production, so as to clarify whether readers obtain political news from one newspaper, and learn about health risks from their friends and acquaintances on other channels such as social media platforms.

Furthermore, the article employs Foucauldian theorizing of risks emphasizing the power of discourse to systematically construct truths around the risk that can inspire individual and societal action. According to the authors, “a discourse of individual obesity risk responsibility” (Brookes and Baker, 2021a: p. 13) is formed in all other sections of national newspapers in the corpus except in the left-leaning broadsheets. Readers are shaped as agents taking responsibility for managing obesity risks by making the right decisions so that obesity is controlled, related health problems will be reduced and children would be healthier. It shows that readers seem to have the final decision-making rights to their own, but in fact, this neoliberal notion has exerted hidden power to have shirked the government's responsibility in obesity risk management. Therefore, we side with the authors that individuals will be made scapegoats if obesity risks occur. At the same time, we still take into account that power in discourse is like a chain or network, which is not totally from the up-bottom direction, but rather in a bottom-up way (Foucault, 1997). Thus, common readers can take societal action to resist the press's influence in this neoliberal era. Additionally, we propose that further research studies be carried out to take account of readers' responses in their counter-discourse to look at if they take up the position framed by the press.

## Conclusion

To sum up, we find this article clearly illustrated and thought-provoking. It demonstrates that potentially fear-inducing rhetoric has been constructed by the UK newspaper coverage of obesity, which stimulates the individual's responsibility in the predominant neoliberal discourses in recent years. Such scarce tactics are employed by many mass media and public health text producers to secure the audience's attention, hoping the fear stimulated in the audience could make them take recommended course of action. This article also points out that this personal and fear-based discursive framing of risk as relevant to obesity may be effective, but the public can be better served by more positive appeals of hope and rewards. Through corpus linguistic methods, the study provides insights and references as to how the certain social psychological phenomenon is induced through discursive strategies in the media.

As obesity risks have attracted the attention of researchers, practitioners, and readers lately, particularly in the context of COVID-19, we strongly suggest that future studies address diverse interests from various contexts. We also propose other important influencing factors in media representation of obesity-related risks in specific social, political, religious, and historical contexts be put under investigation.

## Author contributions

JZ drafted the opinion article. XZ helped JZ to select the commented article, provided insights and suggestions during her writing, and did the revision for the manuscript. All authors contributed to the article and approved the submitted version.

## References

[B1] Atanasova D. Koteyko N. (2017). Obesity frames and counter-frames in british and german online newspapers. Health 21, 650–669. 10.1177/1363459316649764 27207941

[B2] Baker P. (2006). Using Corpora in Discourse Analysis. London: Continuum. 10.5040/9781350933996

[B3] Baker P. Gabrielatos C. McEnery T. (2013). Discourse Analysis and Media Attitudes: The Representation of Islam in the British Press 1998-2009. Cambridge and New York: Cambridge University Press. 10.1017/CBO9780511920103

[B4] Berger A. A. (2016). Applied Discourse Analysis: Popular Culture, Media, and Everyday Life. London: Palgrave Macmillan. 10.1007/978-3-319-47181-5

[B5] Brookes G. Baker P. (2021a). Fear and responsibility: discourses of obesity and risk in the UK press. J. Risk Res. 1–16. 10.1080/13669877.2020.1863849

[B6] Brookes G. Baker P. (2021b). Obesity in the News: Language and Representation in the British Press. Cambridge: Cambridge University Press. 10.1017/9781108864732

[B7] De Pergola G. Silvestris F. (2013). Obesity as a major risk factor for cancer. J. Obes. 13, 291546. 10.1155/2013/291546 24073332 PMC3773450

[B8] Fairclough N. (1995). Media Discourse. London: Edward Arnold.

[B9] Foucault M. (1969). The Archaeology of Knowledge. London: Routledge.

[B10] Foucault M. (1997). “Governmentality,” in Power, ed Rabinow (New York, NY: The New Press), 201–222.

[B11] Fruh S. M. (2017). Obesity: risk factors, complications, and strategies for sustainable long-term weight management. J. Am. Assoc. Nurse Pract. 29, 3–14. 10.1002/2327-6924.12510 29024553 PMC6088226

[B12] Hardie A. (2012). CQPweb – combining power, flexibility and usability in a corpus analysis tool. Int. J. Corpus Linguist. 17, 380–409. 10.1075/ijcl.17.3.04har

[B13] Hastings G. Stead M. Webb J. (2004). Fear appeals in social marketing: strategic and ethical reasons for concern. Psychol. Market. 21, 961–986. 10.1002/mar.20043

[B14] Huang X. Bisiada M. (2021). Is obesity just a health issue? Metaphorical framings of obesity in the people's daily. CADAAD J. 13, 18–40.

[B15] Johnson S. Milani T. M. eds. (2010). Language Ideologies and Media Discourse: Texts, Practices, Politics. London; New York, NY: Continuum.

[B16] Kress G. Van Leeuwen T. (2001). Multimodal Discourse: The Modes and Media of Contemporary Communication. London: Arnold Publishers.

[B17] Kwan S. Graves J. (2013). Framing Fat: Competing Constructions in Contemporary Culture. New Brunswick, NJ; London: Rutgers University Press.

[B18] Lawrence R. G. (2004). Framing obesity: the evolution of news discourse on a public health issue. Harvard Int. J. Press Polit. 9, 56–75. 10.1177/1081180X04266581

[B19] Lupton D. (2013). Risk, 2nd Edn. London: Routledge. 10.4324/9780203070161

[B20] Machin D. Van Leeuwen T. (2007). Global Media Discourse: A Critical Introduction. London; New York, NY: Routledge. 10.4324/9780203007471

[B21] Malik V. S. Willett W. C. Hu F. B. (2013). Global obesity: trends, risk factors and policy implications. Nat. Rev. Endocrinol. 9, 13–27. 10.1038/nrendo.2012.199 23165161

[B22] Sakellariou D. Rotarou E. S. (2017). Access to healthcare for men and women with disabilities in the UK: secondary analysis of cross-sectional data. BMJ Open 17, e016614. 10.1136/bmjopen-2017-016614 28893735 PMC5629679

[B23] Segel C. M. (2011). Childhood Obesity: Risk Factors, Health Effects, and Prevention. New York, NY: Nova Science.

[B24] Talbot M. (2007). Media Discourse: Representation and Interaction. Edinburgh: Edinburgh University Press. 10.1515/9780748630073

[B25] Thurlow C. Mroczek K. (2011), Digital Discourse: Language in the New Media. London; New York, NY: Oxford University Press. 10.1093/acprof:oso/9780199795437.001.0001

[B26] Triandafyllidou A. Wodak R. eds. (2009). The European Public Sphere and the Media: Europe in Crisis. Basingstoke: Palgrave Macmillan. 10.1057/9780230271722

[B27] Wexler B. (2005). Weight in America: Obesity, Eating Disorders, and Other Health Risks, Thomson Gale. Available online at: http://LJ3LE7ZK2E.search.serialssolutions.com/?V=1.0andL=LJ3LE7ZK2EandS=JCsandC=TC0001342823andT=marc (accessed October 09, 2022).

